# Cancer: A Disease of Modern Times?

**DOI:** 10.7759/cureus.74666

**Published:** 2024-11-28

**Authors:** Natasha Nwosu

**Affiliations:** 1 Oncology, University College London Hospitals NHS Foundation Trust, London, GBR

**Keywords:** modern industrialisation, modifiable risk factors, origins of cancer, population health, tobacco smoke

## Abstract

Cancer is a leading cause of mortality worldwide. Despite its prevalence, its origins remain a topic of debate, sparking discussion within the medical and historical professions. It had been feared for centuries, initially perceived as an incurable condition fraught with social stigma. This review details the historical records of cancer, tracing its earliest documented cases in ancient Egyptian and Greek civilizations, and follows the evolution of oncology treatment. Advances in medical technology and screening methods may have led to improved detection of cancer, with conditions that may have gone unnoticed in the past now diagnosed more frequently. Hippocrates and Galen later advanced the understanding of cancer by shifting from supernatural theories to a more scientific approach.

The role of modifiable risk factors is highlighted, as well as the controversy surrounding the concept that cancer is a product of modern times. Environmental influences such as air pollution and tobacco smoke have been identified as carcinogenic, with public health campaigns subsequently developed. Whilst these preventative efforts aim to reduce such risks, evidence suggests that the causes of cancer are likely multi-faceted and, therefore, focusing solely on industrial factors may simplify a complex issue. The discussion remains open as to whether cancer is truly the product of modern industrialisation, or whether its low incidence in the past was due to less effective documentation and understanding. The apparent low prevalence of cancer in ancient times raises questions about the role of carcinogenic environmental factors in modern societies, with a need for continued research to explore its true origins and, therefore, develop effective strategies for prevention and treatment.

## Introduction and background

Cancer is a group of diseases, characterised by uncontrolled growth and spread of abnormal cells. It can develop in several different parts of the body and is influenced by a variety of factors, including genetics, environmental exposures, lifestyle choices, and infections [1]. Over time, cancer has become increasingly recognised as a leading cause of death and this may be due, in part, to the development in management and consequent decreasing prevalence of other causes of mortality. Examples of these include stroke and cardiovascular disease [2], where the decreased prevalence may be related to improved primary prevention strategies, such as NHS health checks. Based on recent trends in global mortality, it is projected that cancer may surpass cardiovascular disease as the leading cause of premature death in most countries over the course of this century [2].

Approximately one in five deaths worldwide is caused by cancer and, considering global cancer statistics produced by the International Agency for Research on Cancer, it is estimated that the burden of cancer will rise significantly over the next 20 years [3]. These statistics used estimates of incidence and mortality for 36 cancers across 185 countries and analysed both the number of new cases and new deaths associated with each of these types of cancer. At 2,261,419 (11.7%), breast cancer produced the largest proportion of new cases and the large proportion of new deaths was associated with lung cancer, i.e., 1,796,144 (18%) of new deaths. The global cancer burden is expected to increase to 28.4 million cases in 2040, with a larger projected increase in developing (64% to 95%) versus developed (32% to 56%) countries; this may be in part due to demographic changes and globalisation [3], with the development of health education particularly in more developed countries, with widespread use of the NHS app for weight management, for example.

In the United Kingdom, a significant proportion of cancers are thought to be related to modifiable risk factors, including smoking, excessive alcohol consumption, obesity, and physical inactivity [4]. Nearly four in 10 cancer cases in 2015 in the UK were proposed to be attributable to known risk factors, with tobacco smoking and obesity thought to contribute the most. Tobacco smoking contributed the largest proportion of attributable cancer cases, accounting for 15.1% of all cases. The 2018 study, undertaken by Brown et al., analysed 10 different cancers and concluded that more than 70% of cancer cases were attributable to known risk factors [4].

This highlights the need for the regular collection of risk factor exposure prevalence data and the fact that prevention efforts are likely to have the largest population-level impact. The study does have some limitations, including the assumption made that the relative risk of cancer associated with each risk factor is consistent across different populations and the fact that the study only includes risk factors with convincing evidence of an association with cancer, therefore not necessarily capturing all potential risk factors. The study also uses data from 2005, which may not reflect current risk factor prevalence or cancer incidence rates. These data limitations are therefore biased towards underestimation of potential risk factors and this is acknowledged by the authors.

Whilst the incidence of cancers is projected to increase, the rate of increase appears to be slowing down and one of the proposed reasons for this is a marked reduction in one of the most significant modifiable risk factors for developing the disease, i.e., tobacco use [5]. Cancer incidence rates are projected to decrease by 0.03% in males and increase by 0.11% in females yearly between 2015 and 2035. This may be due to longer predicted life expectancies in the female population. Whilst most cancer mortality rates are decreasing, thyroid, liver, oral, and kidney cancer are among the fastest-accelerating cancers [5]. This highlights the increasing burden of cancer worldwide and underscores the need for effective prevention and treatment strategies.

## Review

During the 19th and early 20th centuries, cancer was often seen as an incurable and dreaded disease, leading to significant social stigma and fear [6]. This perception has evolved with the development of research and treatment options, although cancer remains a major health concern. The development of surgical techniques in the 17th century marked a significant advancement in oncology, followed by the introduction of radiotherapy and chemotherapy in the late 19th and mid-20th centuries, respectively, further transforming cancer treatment- detailed further below.

The earliest records of cancer date back to ancient civilizations, such as the Egyptians and Greeks, with texts documenting cases appearing to describe malignancies [7]. Historical records suggest that cancer has existed for thousands of years, but the understanding and classification of the disease have evolved. In ancient times, many diseases were not well understood, and cancer may have been misdiagnosed or not diagnosed at all. A record that is widely accepted as the first-ever documentation of cancer was found in the ancient Egyptian Edwin Smith Papyrus, dated 3000 BC, describing tumours and ulcers resembling cancer. It detailed breast cancer, described by the author as an incurable bulge of the tissue [7]. This was followed, in 1500 BC, by the Ebers Papyrus, in which the writer described tumours of the skin, stomach, rectum, and uterus [6]. Hippocrates and Galen contributed significantly to the understanding of cancer, moving away from supernatural explanations to those which were more science-based [6]. Until the influence of Hippocrates took root, cancers were viewed as having supernatural causes and were as such treated with rudimentary methods, including knives, pastes, and salts [8]. Some modern scientists therefore disregard the Egyptian texts as reliable sources of information. Some of these scientists believe the accounts to be descriptions of varicose veins and swellings, rather than tumours [3].

Hippocrates is known to have coined the terms ‘acute’, ‘remission’, ‘chronic’, and ‘relapse’ [9] and introduced the new theory that cancer was to be treated firstly with medicine, and if not successful, for it to be operated on. If cautery was not successful, then the cancer was deemed incurable [6]. Drawing on Hippocrates’ humour theory, Galen, a Roman surgeon, theorised that an excess of black bile caused incurable cancer and ulcers, while curable nonulcerated cancer was the result of an excess of yellow bile [9].

Following on from the ancient Egyptian and Greek records, there were minimal public records of treatments for cancer until the 17th century, when physicians such as Marco Aurelio Severino and Johannes Scultetus produced widely circulated publications detailing predominantly surgical oncological techniques, as well as, in the case of Severino, information about the classification of cancer. After this came the period of enlightenment and a record of the first modernised cancer surgery techniques, produced by the well-renowned Dr John Hunter [10]. From then, came publications about the role of radiotherapy, born in 1895 with the use of X-rays to prevent relapses of breast cancer and then in 1903, the use of radioactive substances to treat malignancies [11].

Intravenous chemotherapy’s role in cancer treatment (first recognised for its role in treating leukaemia) came out in 1947 [12] and immunotherapy, though largely considered to be a recent development in the treatment of cancer, can arguably trace its roots back to the late 19th century where Dr Colen, who noted multiple cases of spontaneous cancer remission in patients with concomitant bacterial infections, showed the power of harnessing the body’s own immune system to mount a response against malignancies [13]. At the time, however, he did not hold a significant position in the medical community and therefore, the methods of surgery and radiotherapy were much preferred. The crucial role of T cells did not come to the forefront of medicine until 1967 [13], and since then, the role of immunotherapy has only continued to grow. These are significant historical milestones in the understanding and treatment of cancer, tracing its evolution from ancient times to modern oncology.

In 2010, Professors Michael Zimmerman and Rosalie David conducted extensive research into the origins of cancer, by investigating medical reports of human and non-human remains. This included thousands of Greek and Egyptian remains, as well as the literature written at the time [13]. Zimmerman was the first to identify cancer from Egyptian remains that were around 1700 years old. He found histological evidence of colon cancer and after this discovery, the scientists found further cases of tumours by analysing rehydrated tissues of fossils and analysing ancient medical texts, the majority of which turned out not to be malignant, indicating a significantly lower incidence of cancer compared to modern populations [13].

Soon after this discovery, Zimmerman released a statement, in which he used his findings as evidence that disease-causing factors are predominantly limited to modern society and more specifically, post-Industrial Revolution, after which the incidence of cancer increased almost exponentially [14]. The authors conclude that cancer is a modern disease caused by modern lifestyle issues, such as tobacco use and pollution resulting from industrialisation. The study found little evidence of cancer in the ancient skeletons and mummies or through analysis of ancient texts and literature from Egypt and Greece, leading the authors to suggest that cancer is a disease of modern times.

However, this conclusion has been criticised by cancer researchers, who argued that there are many natural causes of cancer and that the study's findings were not surprising, given the age of the skeletons and mummies. They pointed out several limitations of the study, including the fact that most of the skeletons and mummies were of people who died before the age of 50. They highlighted the fact that ageing is a major cause of cancer, suggesting that in the hypothetical examination of somebody to have passed away before the age of 50 in modern society, there would similarly also be very few cases where cancer was found [15]. They expressed concern with the notion of blaming industrialisation for cancer, as it may make people feel helpless and divert attention from the many potential behaviour changes to reduce their risk of cancer, such as quitting smoking, exercising more, drinking less, and eating more healthily.

A Cancer Research UK advocate spoke out against David’s statement, by highlighting some of cancer’s natural causes, including ultraviolet light rays, natural radiation, and viruses, such as human papillomavirus (HPV), linked to cervical cancer for example [15]. Over time, this has seen a decreased incidence, likely due to vaccination and screening programmes, such as the introduction of the HPV screening programme, which is now also offered to male patients. The author of the book ‘The Biology of Cancer’, Robert A. Weinberg, spoke out against the evidence, by bringing attention to advances in medical technology. He hypothesised that, due to the modern diagnostic techniques available today, it is far easier for us to identify the cancers, but that this in no way suggests that the cancer was any less common, but rather that the people studied died of other more common causes [16].

One of the significant factors contributing to the perception that cancer is a modern disease is increased life expectancy. As people live longer, they are more likely to develop cancer, as the risk of many types of cancer increases with age [17]. Joachim Schüz, head of the International Cancer Research Centre in France, emphasised that if the tissues of under 50s were to be analysed today, the majority would be found to be cancer-free [15]. This point of view was shared by Dr John Glaspy, an oncologist based at the University of California, who highlighted that nowadays, cancer is rare among those under 30 and since a large proportion of the Greek and Egyptian populations did not live past this age, this may be the reason why cancer was so seemingly rare [18].

Zimmerman and David ascertained that in this population, people typically lived for 25 to 50 years, depending on their social class. Despite the generally lower life expectancy, which came about as a result of higher infant mortality rates and poorer infection control [19], some upper-class individuals lived long enough to acquire conditions such as arthritis and atherosclerosis, both of which are related to age [14]. Zimmerman therefore argued that there is no reason why cancer would not have been discovered too.

Zimmerman and David used the lack of findings of bone carcinomas amongst younger individuals as further evidence for their theory [14]. Schüz and cell biologist Mel Greaves countered this point of view by arguing that the sample size was too small to warrant this conclusion; the incidence of childhood osteosarcomas is one in 10,000, therefore it may not be valid to conclude this when just a few thousand samples were taken [16]. It could be hypothesised that so few cases of cancer may have been found because the tumours did not stand the test of time. Whilst it may be widely believed that the damage that tumours cause to cell tissue may cause them to deteriorate more quickly, Zimmerman suggested that the tissue may actually be better preserved than non-affected tissue.

To investigate the significance of the size of the sample used during the research, paleopathologist Tony Waldron carried out his own research. Using the data from 20th-century mortality reports, he estimated that the incidence of cancer should amount to less than 2% amongst the male remains and should range from 4% to 7% amongst the females. When researchers in Munich tested this, the results matched his hypothesis; the number of cases of cancer was no fewer than expected [16].

In 2016, data released by the charity ‘Children with Cancer UK’ showed the incidence of cancer amongst young people to be rising from 10 to 16 in 100,000, and scientists suggest that even adjusting for improved screening, it is most likely due to environmental factors, i.e., modern factors that were not present years ago. According to Dr Denis Henshaw, scientific advisor for the charity, air pollution accounts for 40% of the increase, while the remaining factors include radiation exposure from hospital scans, childhood obesity, pesticides, and solvents [19]. Analysis of government statistics by researchers at the charity Children with Cancer UK found that there are, on average, 1,300 more cases of cancer per year. The study found that the number of children diagnosed with cancer has risen 40% over 16 years since 1998, with the rise in cases estimated to cost the NHS an extra £130 million a year compared with 16 years ago [20].

The rise is most apparent in teenagers and young adults aged between 15 and 24 years, where the incident rate has risen from around 10 cases in 100,000 to nearly 16. Diagnoses of colon cancer among children and young people have risen by 200% since 1998, while thyroid cancer has doubled. Researchers say that although some of the rise can be explained by improvements in cancer diagnoses and more screening, the majority may be caused by environmental factors. More than 4,000 children and young people are diagnosed with cancer every year in Britain, and cancer is one of the leading causes of death in children aged one to 14 years. Dr Denis Henshaw, professor of human radiation effects at Bristol University, and the scientific adviser for Children with Cancer UK, suggested air pollution to be the biggest culprit, accounting for approximately 40% of the rise [20].

Smoking during and after pregnancy, exposure to magnetic fields from power lines and radiation from CT scans and X-rays, circadian rhythm disruption through excessive exposure to bright light at night, pesticides, and solvents ingested or inhaled during pregnancy and potentially radiation from mobile phones have also been thought to contribute. The article does note the difficulty of avoiding many of the environmental factors contributing to the rise in cancer diagnoses, also suggesting that lifestyle changes such as allowing children to attend nursery to boost their immune system, avoiding shift work, and eating healthily can help to reduce the risk of cancer [20].

Multiple environmental factors, notably tobacco smoke, have been confirmed to be carcinogenic. The first tenuous link between smoking and lung cancer was made in 1912 when Isaac Adler noted the sudden increase in the incidence of the disease. After the correlation was confirmed in the mid-1900s, public health campaigns to reduce smoking soon began. It has recently been revealed that for every three to four million cigarettes smoked, there is one death caused by lung cancer, and around 95% of the deaths are entirely preventable [21]. It is now a leading cause of death, whereas, before the 20th century (before cigarettes), medical students were told that they would be lucky to see more than one patient with the disease in their lifetime [21].

The WHO's International Agency for Research on Cancer (IARC) released information suggesting that ‘Roundup’, one of the most widely used herbicides, contained glyphosate, a substance that was ‘probably’ carcinogenic to humans. In the UK, the Soil Association called for a ban on the use of glyphosate by farmers, particularly in conjunction with genetically modified crops, arguing that the use of the weedkiller has risen by 400% in the last 20 years in UK farming and is one of the three pesticides regularly found in routine testing of British bread. The report did, however, add that in the areas around people’s homes, the levels of the chemical were not worryingly high and the German Federal Institute for Risk Assessments (BfR) found limited evidence of carcinogenicity in mice exposed to glyphosate and recommended its re-approval, with a relaxation in the acceptable daily intake from 0.3 to 0.5 mg per kilogram of body weight per day.

Their report was, however, criticised for relying heavily on unpublished industry reports [22]. Soon afterwards, a further report was published, deeming the weed killer safe to use and ‘Monsanto’, the weed killer company, accused the WHO of having conflicts of interest when releasing the report [22]. This demonstrates how, even in modern times, the true carcinogenic impact of risk factors, environmental or otherwise, is still under discussion and difficult to prove definitively.

So far, Zimmerman and David’s study, the principal one in this field, has received more criticism than support, but David acknowledged this and emphasised that the purpose of the study was not to blame cancer on modern living but rather to suggest that it may be part of the cause. It could also be argued that it sparked an important debate amongst academics. Just as with numerous other health conditions, the origins are not always straightforward to identify but our understanding evolves alongside society; with the evolvement of society comes changes in behaviour, which can impact our relationship with cancer, be this positively or negatively.

## Conclusions

Whilst cancer is not necessarily exclusively a disease of modern times, the factors contributing to its prevalence have changed significantly. Whilst it has existed throughout human history, the modern lifestyle, increased life expectancy, and improved detection methods have influenced our understanding and the rates of diagnosis. Thus, cancer can be seen as a disease that has likely existed historically, albeit less frequently identified due to shorter life expectancies and limited diagnostic techniques. It is now better understood due to advancements in medicine and changes in society. The dramatic rise in cancer cases in the 20th and 21st centuries could be attributed to factors such as increased tobacco use, industrialisation, and modern lifestyle changes, including diet, sedentary behaviour, and environmental pollution. Advances in medical technology and oncological treatment have improved the detection and treatment of cancer, making it a more prominent diagnosis in modern times.

Cancer is an incredibly complex disease whose origin is difficult to define. The events surrounding the ‘Roundup’ herbicide go to show that the ‘cancer scare’ does exist, so much so that information linking carcinogens to environmental factors may sometimes be published prematurely, often in the interest of scientific research and increasing public awareness. A deeper understanding of cancer's historical and modern contexts, the evolution of its treatment, and the impact of modifiable risk factors on its incidence can help to inform prevention efforts and improve treatment outcomes. The discussion surrounding cancer's origins is crucial for developing effective public health strategies and interventions. This review highlights the need for continued research into the origins of cancer, particularly in understanding how historical and modern factors interact. This may include investigating the long-term effects of lifestyle changes on cancer incidence, exploring the role of environmental pollutants and their impact on health and enhancing diagnostic methods to better understand cancer prevalence in historical and modern populations.

## References

[1] Sarkar S, Horn G, Moulton K, Oza A, Byler S, Kokolus S, Longacre M (2013). Cancer development, progression, and therapy: an epigenetic overview. Int J Mol Sci.

[2] Bray F, Laversanne M, Weiderpass E, Soerjomataram I (2021). The ever-increasing importance of cancer as a leading cause of premature death worldwide. Cancer.

[3] Sung H, Ferlay J, Siegel RL, Laversanne M, Soerjomataram I, Jemal A, Bray F (2021). Global Cancer Statistics 2020: GLOBOCAN estimates of incidence and mortality worldwide for 36 cancers in 185 countries. CA Cancer J Clin.

[4] Brown KF, Rumgay H, Dunlop C (2018). The fraction of cancer attributable to modifiable risk factors in England, Wales, Scotland, Northern Ireland, and the United Kingdom in 2015. Br J Cancer.

[5] Smittenaar CR, Petersen KA, Stewart K, Moitt N (2016). Cancer incidence and mortality projections in the UK until 2035. Br J Cancer.

[6] Risse GB (1989). The dread disease: cancer and modern American culture. Med Hist.

[7] Hajdu SI (2011). A note from history: landmarks in history of cancer, part 1. Cancer.

[8] Hajdu SI (2004). Greco-Roman thought about cancer. Cancer.

[9] Hajdu SI (2017). Pathfinders in oncology from the end of the Middle Ages to the beginning of the 19th century. Cancer.

[10] Cohen B (1993). John Hunter, pathologist. J Royal Soc Med.

[11] Vujošević B, Bokorov B (2010). Radiotherapy: past and present. Arch Oncol.

[12] Scott RB (1970). Cancer chemotherapy--the first twenty-five years. Br Med J.

[13] Dobosz P, Dzieciątkowski T (2019). The intriguing history of cancer immunotherapy. Front Immunol.

[14] David AR, Zimmerman MR (2010). Cancer: an old disease, a new disease or something in between?. Nat Rev Cancer.

[15] Charles Q (2024). Cancer is a man-made disease, controversial study claims. Choi: Cancer Is a Man-Made Disease, Controversial Study Claims.

[16] Rozhok AI, DeGregori J (2016). The evolution of lifespan and age-dependent cancer risk. Trends Cancer.

[17] Zimmerman MR (2011). The analysis and interpretation of mummified remains. A Companion to Paleopathology.

[18] (2024). Deccan Herald. Antiquity of cancer. http://www.deccanherald.com/archives/antiquity-cancer-2385482.

[19] Coghlan A (2024). Briefing: cancer is not a disease of the modern world. https://www.newscientist.com/article/dn19591-briefing-cancer-is-not-a-disease-of-the-modern-world/.

[20] Knapton S (2024). The Telegraph. Modern life is killing our children: cancer rate in young people up 40 per cent in 16 years. https://www.telegraph.co.uk/science/2016/09/03/modern-life-is-killing-our-children-cancer-rate-in-young-people/.

[21] Proctor RN (2012). The history of the discovery of the cigarette-lung cancer link: evidentiary traditions, corporate denial, global toll. Tob Control.

[22] Neslen A, Levitt T (2024). Weedkiller suspected of causing cancer deemed 'safe'. https://www.theguardian.com/environment/2015/jul/15/weedkiller-suspected-of-causing-cancer-deemed-safe.

